# Assessment of the perception of vertical subjectivity in children born preterm

**DOI:** 10.1007/s00431-023-04863-y

**Published:** 2023-03-01

**Authors:** Laura Riera-Tur, Manuel Lubián-Gutiérrez, Isabel Benavente-Fernández, Simón Lubián-López, Antonio J. Martín-Mateos, Alfonso M. Lechuga-Sancho

**Affiliations:** 1 Department of Otolaryngology, Puerta del Mar University Hospital, Av. Ana de Viya 21, CP: 11009 Cádiz, Spain; 2 Biomedical Research and Innovation Institute of Cádiz (INiBICA) Research Unit, Puerta del Mar University, Cádiz, Spain; 3 Department of Paediatrics, Puerta del Mar University Hospital, Cádiz, Spain; 4 Division of Neonatology, Department of Paediatrics, Puerta del Mar University Hospital, Cádiz, Spain; 5 Area of Paediatrics, Department of Child and Mother Health and Radiology, Medical School, University of Cádiz, Cádiz, Spain

**Keywords:** Balance, Prematurity, Smartphone, Vertical subjective visual test

## Abstract

Children born preterm have increased rates of paediatric mortality and morbidity. Prematurity has been associated with impaired visual perception and visuo-motor integration. The alteration of the perception of verticality translates into alterations of the vestibular system at central and/or peripheral level, which may manifest itself in symptoms such as imbalance, dizziness or even vertigo. The aim of this study was to compare subjective visual vertical (SVV) test scores in children born preterm with those of children born at term at ages between 7 and 10. One hundred ten children with no neurodevelopmental disorder of 7 to 10 years of age were studied using a mobile application on a smartphone attached to a wall by means of a rotating plate. The SVV test was compared between two groups: a group of 55 preterm children (53 very preterm children born under 32 weeks of gestational age and 2 preterm with very low birth weight) and another group of 55 children born at term (after 37 weeks of gestational age). The SVV results were analysed for comparison with respect to prematurity, sex and age. We found no significant differences in the SVV study in the comparison between preterm and term children. In addition, no significant differences were observed regarding sex or age between 7 and 10 years.

*  Conclusion*: We found no alterations in the perception of vertical subjectivity in children between 7 and 10 years of age, with antecedents of very preterm birth and/or very low birth weight.

**Table:** 

**What is Known:**
• *The different studies published so far suggest the existence of balance disorders in premature children, although in most of these studies the children are examined at an age when the vestibular system is not mature and with non-specific tests for the study of the vestibular system.*
**What is New:**
• *We compared the results of the subjective visual vertical (SVV) test in a group of 55 preterm children (53 very preterm children born under 32 weeks of gestational age and 2 preterm with very low weight at birth) and in a group of 55 children born at term (after 37 weeks of gestational age), at the ages of 7 to 10 years and observed no differences.*
• *We conclude that, if there had been any vestibular alterations due to very premature birth, these must have been compensated by the age of 7.*

## Introduction

A preterm birth is one that occurs before 37 weeks of gestation. Preterm birth rates in Europe are between 5 and 9%, with an increasing trend in recent years, due to the progressive increase in artificially conceived pregnancies, and an increased number of indications for preterm birth [1]. Preterm birth increases the risk of paediatric mortality and morbidity. In developed countries, there has been a decrease in mortality due to advances in perinatal care; however, high morbidity persists mainly due to neurodevelopmental disorders, particularly in very low birth weight and or very preterm children (gestational age below 32 weeks and/or under 1500 g of weight at birth) [2–4].

Neurodevelopmental disabilities in preterm infants affect many areas including impairments in visual perception and visual-motor integration, cognitive and behavioural impairments. These alterations have an impact on the adaptive capacities that children acquire in the long term, involving deficits in areas such as balance. Balance may be affected by alterations in the development of other areas that occur in premature children, such as alterations in motor development [2, 3, 5, 6].

The maintenance of balance and postural control is a multifactorial task that depends on visual, proprioceptive and vestibular inputs which are integrated at the central nervous system. During foetal life until birth at full term, all the structures that will play an essential role in this function are formed. Dziuba et al. argue that the shortening of gestation has an adverse influence on balance in preterm infants [7, 8], but the role of vestibular alterations has been scarcely studied in this population. After birth, the correct development of these areas and the acquisition of compensatory resources will play a crucial role in the acquisition of balance and postural control.

During childhood, there is maturation of the visual, vestibular and somatosensory systems, as well as the development of their integration at the central level, necessary to achieve postural control. In children under 4 years of age who meet all developmental milestones without alterations, there is a visual dependence necessary for the maintenance of posture. This is due to an immaturity of the visual, vestibular and somatosensory systems, and incomplete integration of vestibular and somatosensory inputs at the central level. By the age of 7 years, children are able to make postural adjustments similar to those of adults, which indicates that at this age the maturity of these systems has been reached [7, 22]. Therefore, in addition to the need for patient collaboration in performing vestibular tests, most vestibular studies in children are carried out from this age onwards.

The subjective perception of verticality is essential for the maintenance of balance and for standing upright. Our “verticality sensor” is triggered after receiving an image on the retina, activating the graviceptic system, starting from the vestibular system in the otolithic organs and making connections in the vestibular nuclei, cerebellum, thalamic regions and cerebral cortex [9, 10].

The subjective perception of verticality is explored by asking the patient to correctly orientate a vertical line or an object. There are many devices available for carrying out subjective visual vertical (SVV) tests and some of them are very complex systems. Thanks to technological developments in recent years, our group and others have developed simple and inexpensive devices to perform SVV tests, including mobile applications that are suitable for adult and paediatric populations [11–15]. Specifically, the SVV test using a smartphone attached to a wall-mounted turntable has proven effective and reliable for the detection of vestibular pathology [13].

The aim of this study is to assess, by means of a smartphone attached to a turntable, whether there is an alteration in the perception of verticality in children aged 7 to 10, with history of premature birth.

## Methods

We compared the results of the SVV test in two groups of children with ages ranging from 7 to 10 years. The control group consisted of children born at term and the patient group consisted of children with a personal history of very preterm birth and/or very low birth weight.

### Patients

The study includes 110 children with no neurodevelopmental disorder, 55 preterm and 55 born at full term. The study population consisted of infants born between June 2012 and June 2015 who were admitted to the neonatal intensive care unit of the Hospital Universitario Puerta del Mar. Recruitment was consecutive sampling from infants who were periodically seen in the outpatient clinic, between June 2021 and December 2022. All cases were preterm children, born before 32 weeks of gestation and/or weighted less than 1500 g at birth (53 patients were very preterm and 2 patients were born at 34 weeks but weighted less than 1500 g at birth). The control group were children born between 2012 and 2015, with a gestational age of at least 37 weeks and adequate weight for gestational age, who volunteered to participate in the study as healthy volunteers.

The demographic characteristics of the studied patients are shown in Table 1 and the clinical characteristics of the premature group in Table 2. The two cases of not very preterm correspond to very low weight preterm infants.

**Table 1:** Demographic characteristics of patients

	**Preterm**	**Controls**	***p* value**
Sex			0.84
Boys	27 (51%)	26 (49%)	
Girls	28 (49%)	29 (51%)	
Age			0.98
7 years	6 (11%)	7 (13%)	
8 years	12 (22%)	11 (20%)	
9 years	22 (40%)	16 (38%) 49	
10 years	15 (27%) 48.5	16 (29%) 51.5	
Average age	8.84 ± 0.96	8.84 ± 0.99	
Middle age	9 years	9 years

**Table 2:** Clinical characteristics of the premature group

	**Mean SD**	**Minimum–maximum**	**Categories: frequency (percentage)**
Gestational age	29.5 ± 2.50 weeks	25–34 weeks	Very preterm: 53 (96.4%) Preterm: 2 (3.6%)
Weight	1335 ± 410 g	630–2345 g	Low birth weight: 14 (25%) Very low birth weight: 29 (53%) Extremely low birth weight: 12 (22%)
Length	38.40 ± 3.80 cm	31.50–45.00 cm	
Head circumference	2775 ± 2.40 cm	23.5–33.00 cm	
Delivery			Eutocic: 7 (12.7%) Caesarean: 46 (83.6%) Vacuum-assisted: 2 (3.6%)
Number of births			Only: 26 (47.3%) Twins: 26 (47.3%) Triplets: 3 (5.5%)

*SD* standard deviation

The sample size has been calculated with a confidence level value of 0.05 and a study power of 0.80, for a standard deviation of 2.3 in both groups and a significant mean difference of 1.25 between both groups.

Inclusion criteria for participation in the study were to have an age between 7 and 10 years, understanding of the test to be performed and accepting voluntarily to participate, with informed consent of parents or legal guardians. Exclusion criteria were major congenital malformations, chromosomal abnormalities, congenital infections, acute ear infections, severe visual disturbances, strabismic amblyopia and inability to understand or cooperate with the study test.

### Study test

The subjective visual vertical test was performed by means of a validated mobile application using a smartphone placed on a rotating plate anchored to the wall [13].

**Fig. 1:**
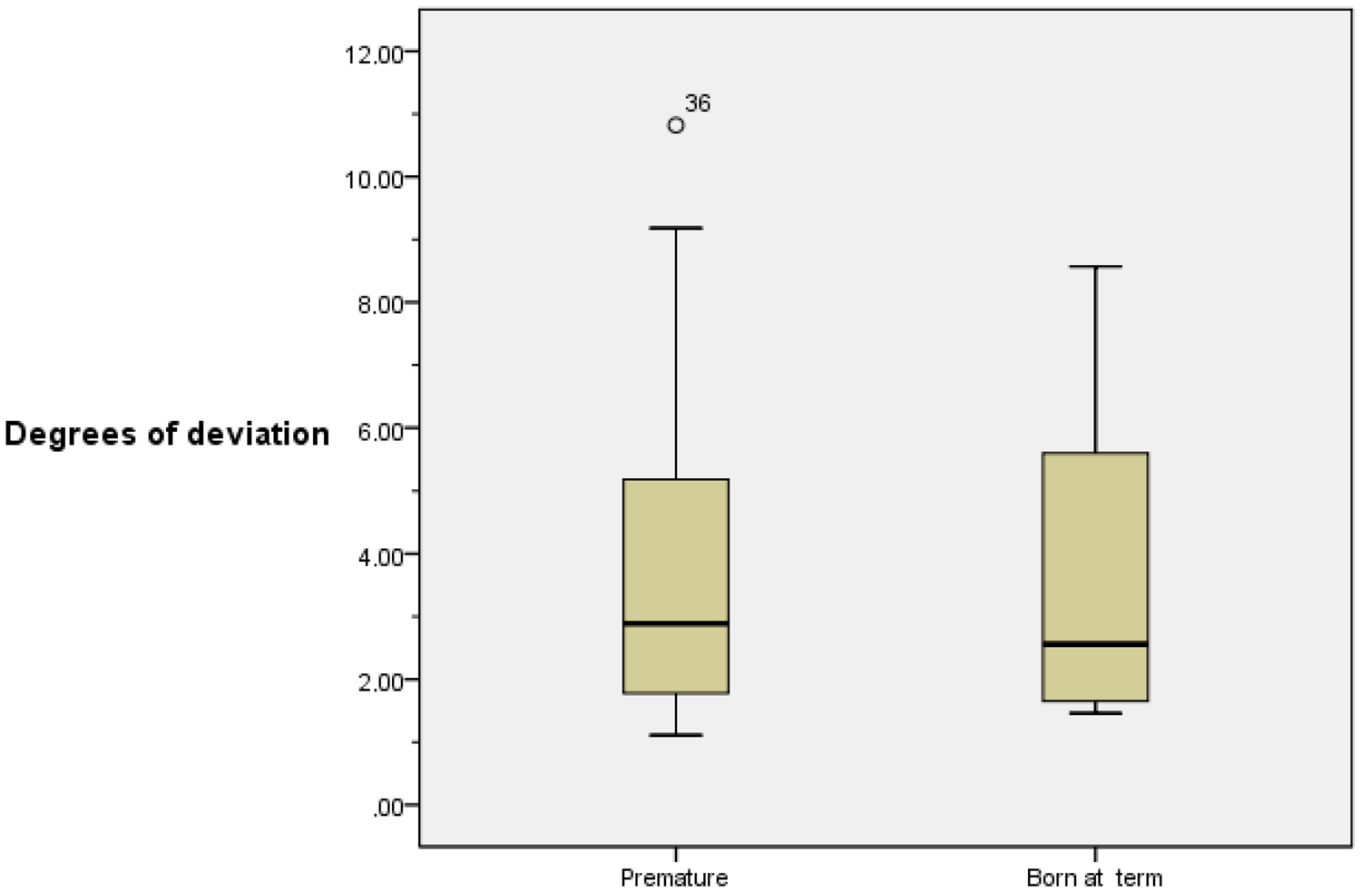
Boxplot. Degrees of deviation (premature and born at term)

**Fig. 2:**
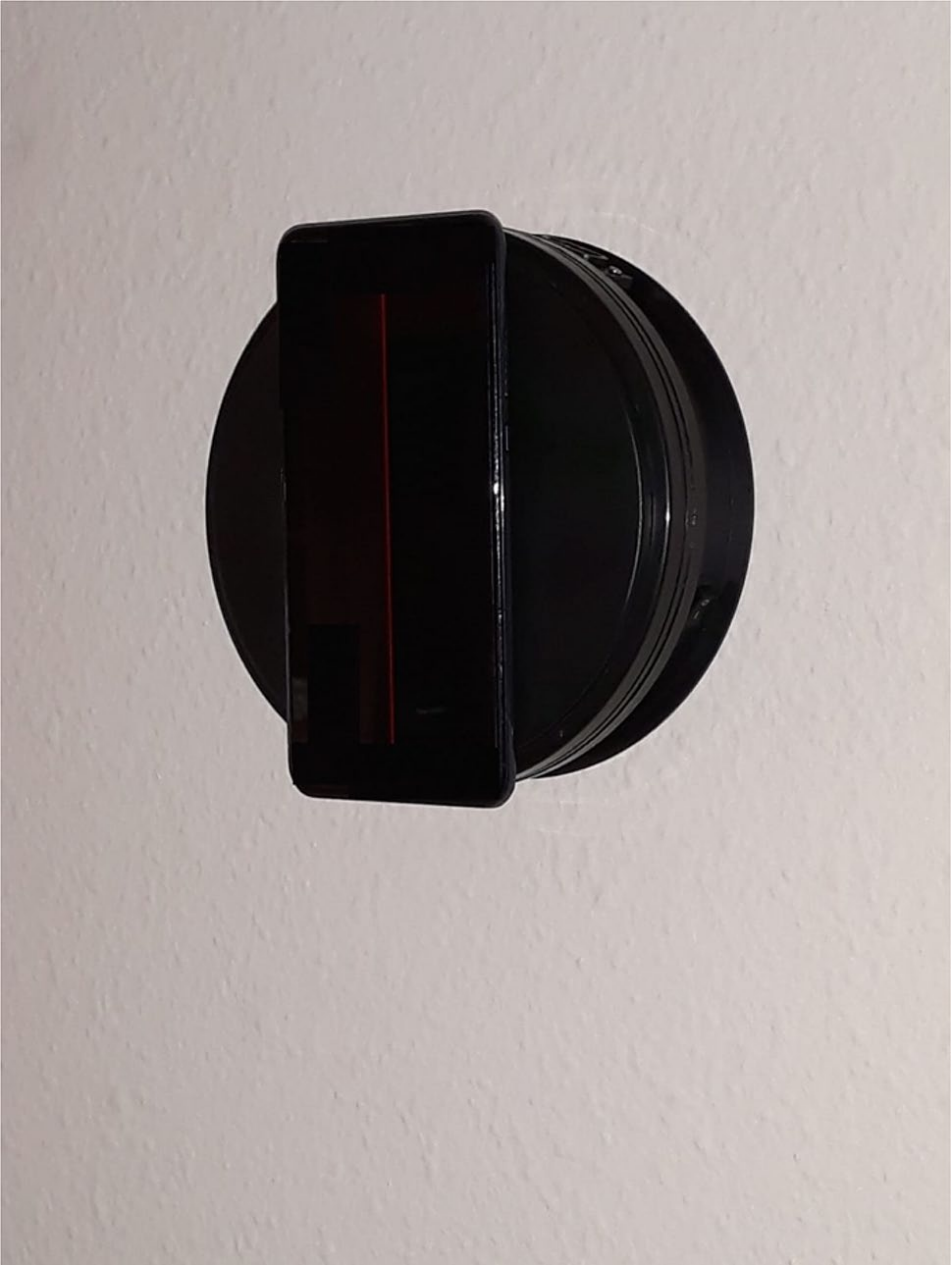
SVV test

Participants sat on a backless bench 1 m away from the device. Participants were asked to focus on a mobile device with a luminous line on the screen against a dark background. The device consists of three elements, a fixed plate on the wall, a smaller plate hinged to the previous one with the ability to rotate 360° and an anchor for the mobile device. To carry out the test, an expert examiner places the device at a starting point where the luminous line is observed at 45° to the real vertical, and then rotates the device clockwise or counterclockwise. The examiner moves the device slowly at a constant speed and the patient indicates to stop the moment when he/she perceives the line to be in vertical position. Once the line is static, the patient is allowed to tell the examiner to move it clockwise or counterclockwise if he/she needs to correct the position of the line until it is exactly vertical (according to his/her perception of verticality). After stopping the device, the degrees of deviation from the actual vertical are recorded (Figs. 1 and 2).

Three measurements were taken on each direction (clockwise and counterclockwise). To avoid the effect of visual accommodation to light, the recording of the first measurement of each direction was discarded. The results were automatically recorded in the database of the mobile device. The SVV test examiner was unaware of the child’s history of prematurity or not, as well as of the results obtained at the time of the scan, which were automatically and blindly stored in an internal database of the smartphone.

Other study variables in the premature group are gestational age, weight, length, head circumference, delivery characteristics and number of births.

The study was carried out at a regional referral hospital after being approved by the institution’s ethics committee.

After approval of the study by the ethics committee, testing is performed on all participants in a single visit.

### Statistical analysis

After inspecting and data scrubbing, statistical analysis was carried out using SPSS v25.0. Firstly, a univariate descriptive analysis was carried out by calculating frequencies for categorical variables and measures of central tendency and dispersion for quantitative variables.

Because the SVV test results did not follow a normal distribution (as determined by the Kolmogorov–Smirnov test), in order to statistically analyse whether there are differences according to prematurity, sex and age, non-parametric tests were used for the analysis (Mann–Whitney *U* test and Kruskal–Wallis test). A *p* value < 0.05 was considered significant.

Multivariable analysis was performed using multiple linear regression including age, sex and study groups (premature to full-term) with the response variable of the SVV test. In the premature group, the multivariate multiple regression analysis was performed with age, sex, gestational age, weight, length, head circumference, delivery characteristics and number of births with the response variable of SVV test.

## Results

The demographic characteristics of the patients studied are shown in Table 1. The mean age for the preterm group was 8.84 ± 0.96 and for the term group it was 8.84 ± 0.99. There were no significant differences in the demographic variables between the two groups.

We found no significant differences between groups at the SVV test outcome measures (Table 3).

**Table 3:** SVV test results in preterm and term infants and children

	**Preterm**	**Controls**
Mean ± SD	3.60 ± 2.26	3.65 ± 2.30
Median	2.89	2.56
Range	9.71	7.11
Mid-range	5.95	5.02
Interquartile range	3.62	4.58
95% CI of the mean	3–4.21	3.03–4.27
P*	0.98	

*SD* standard deviation, *CI* Confidence interval

*Mann–Whitney *U* test

**Fig. 3:**
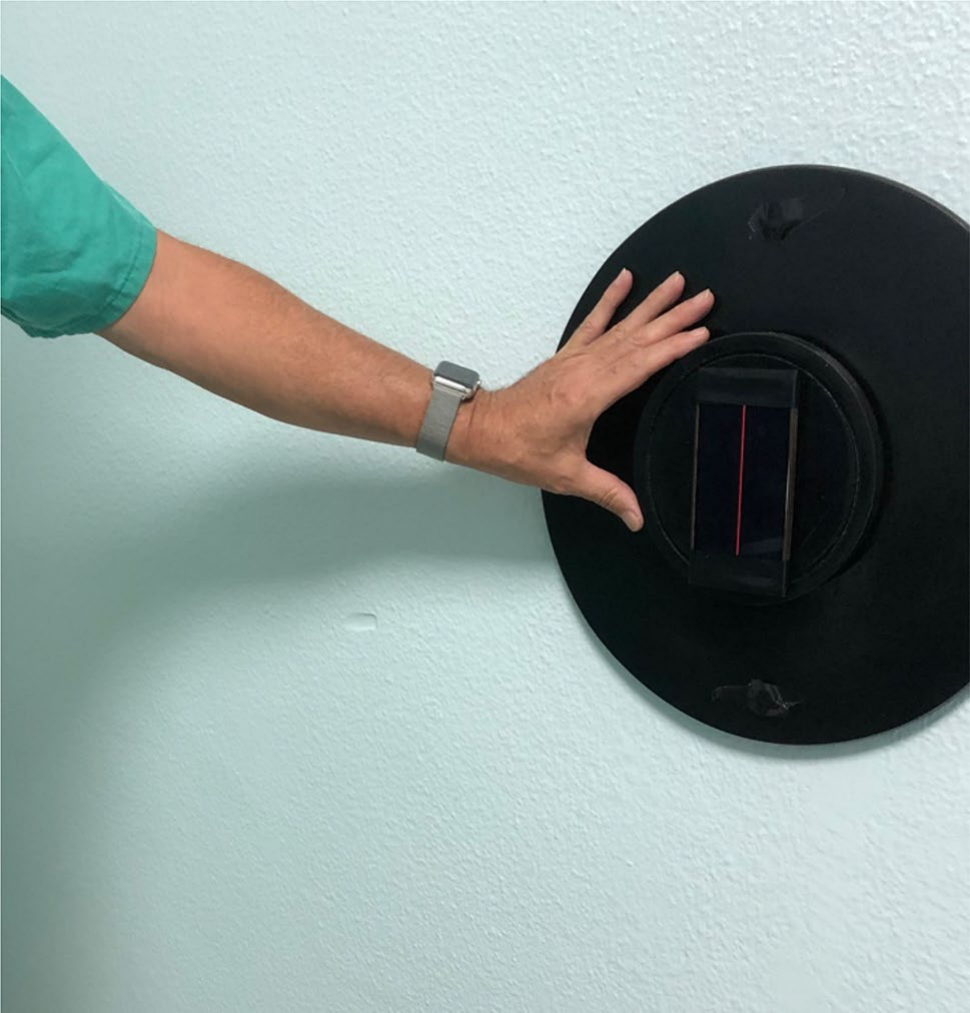
SVV test performance

The box plot shows that the deviations from the median to the fourth quartile are more dispersed in both groups. There is only one extreme value (10.8) in the premature group (Fig. 3).

Neither did we find any significant differences between sex or age and SVV outcome measures (Tables 4 and 5).

**Table 4:** SVV test results in degrees by sex

	**Boys**	**Girls**
Mean ± SD	3.59 ± 2.06	3.66 ± 2.46
Median	3.2	2.41
Range	7.38	9.71
Mid-range	4.88	5.96
Interquartile range	3.22	4.11
95% CI of the mean	3.02–4.16	3–4.31

*SD* standard deviation, *CI* confidence interval

*Mann–Whitney *U* test

**Table 5:** SVV test results according to age

	7 years	8 years	9 years	10 years
Mean ± SD	3.98 ± 2.46	4.42 ± 2.24	3.01 ± 1.92	3.85 ± 2.55
Median	2.67	4.09	2.23	3.41
Range	6.45	6.87	7.46	9.42
Mid-range	4.83	4.27	4.84	6.11
Interquartile range	4.36	3.67	1.82	4.48
95% CI of the mean	2.49–.46	3.30–5.24	2.42–3.60	2.92–4.8
P*	0.129			

*SD* standard deviation, *CI* confidence interval

*Kruskal–Wallis test

In the multivariable analysis using multiple regression including age, sex and study groups (premature or full-term) with the response variable of the SVV test, no significant relationship was observed with any of the variables. In the same way, in the multivariable analysis of the premature group, no significant relationship was observed between the SVV test and any of the variables studied (age, sex, gestational age, weight, length, head circumference, delivery characteristics and number of births) (Fig. 3).

## Discussion

Our study shows that there are no differences between very preterm and full-term infants in SVV test scores in the overall age group of 7 to 10 years, suggesting that any earlier deterioration may have been offset. Due to the sample size of the age ranges, especially at 7 years, we cannot accurately define the lower limit of normalisation.

Balance is a multifactorial task dependent on the central nervous, neuromuscular and visual systems, in which the vestibular system plays a fundamental role. The development of these systems is progressive. Neurodevelopmental disturbances in children with a history of prematurity may lead to a delay in the development of these abilities. However, by the age of 2–3 years, this delay should be compensated for and children should have the same abilities as those born at term [7, 16].

Several studies have shown balance disorders in preterm infants, but these have been assessed using the so-called factorial validity of the Movement Assessment Battery for Children-2nd edition (MACB-2) [7, 8, 17]. This assessment of motor development is by means of a battery of tests, one of which consists of an exercise aimed at assessing balance by means of the child’s ability to maintain stability on one foot [18]. With the one-leg-balance test, balance is evaluated altogether with the participation of the vestibular, visual, proprioceptive and central nervous systems. Therefore, the MACB-2 is not a specific test for assessing vestibular function; balance is assessed through non-specific exercises where other skills such as agility and coordination, which depend more on proprioceptive sensitivity and motor function, could be assessed [8]. With the SVV, the aim is to evaluate the vestibular system in isolation. For its execution, visual support is cancelled by turning off the lights in the room and the patient is seated on a bench without a backrest to suppress proprioceptive afferents as much as possible. In this way, we manage to evaluate only the vestibular system, providing information on its integrity at both central and peripheral levels.

Posturography is a diagnostic test that assesses the patient’s degree of stability and the degree of involvement of each system (vestibular, visual and proprioceptive) in the observed balance disturbance [19]. The results of studies performed with premature children using posturography are in line with our findings. Rodríguez Fernández et al. [8] compared balance in a group with a history of prematurity and another group of children born at term, between 7 and 10 years of age. They carried out the study using the MACB-2 test and posturography. With MACB-2, they found differences which were not reproduced in the posturography assessment [7]. Kluenter et al. also observed no differences between similar groups of children with a history of prematurity and those born at term assessed by posturography at 7 years of age. They suggest that these alterations occur in the first years of life, and by the age of 7, they are compensated for [20].

There are very few studies that have made an assessment of vertical subjective perception in children with a history of prematurity. Bucci et al. in 2015 conducted a study comparing two groups: patients with a history of prematurity of less than 32 weeks and those born at term. They examined both groups when they reached the age of 3 years and found more variable and less accurate SVV test values in the first group than in the second group [21]. This difference with our results may be due to the age at which the examination was carried out. The vestibular system does not reach maturity until the age of 7 years, so the results obtained by analysing 3-year-old children using SVV could be due to a delay in maturation in children with a history of prematurity that could be corrected once maturation of the vestibular system is reached [7, 21, 22]. In a different study, Bucci et al. in 2017 analysed children between 4 and 6 years of age using posturography, and observed differences between the two groups, which could also be due to this fact [23].

The SVV study, unlike MACB-2 or posturography, allows us to assess vestibular function at peripheral and central levels independently of other systems such as proprioceptive. Furthermore, the method used in this study is a simple, convenient and easy test that is very well accepted by paediatric patients given the involvement of technology with a mobile device and the game-like challenge it represents.

Regarding age, our study finds no differences in the results between 7 and 10 years of age, which is in line with the results obtained by Tringali et al. in 2017, who found no differences in SVV between 4 and 9 years of age [24].

## Limitations

Our study was conducted in patients without known neurological disorders after careful clinical evaluation and follow-up. Patients with known neurological disorders were excluded from the study, so we cannot rule out a higher prevalence of balance disorders in preterm infants with neurological affections.

Children were studied in the range between 7 and 10 years of age, so we cannot rule out a greater deterioration of vertical subjective visual disturbances before this period or a slower development from the age of 11 years onwards towards adolescent patterns. Besides, this is a transversal study, without follow-up, which would be desirable, and performed from earlier ages, as soon as collaboration were possible, which is facilitated by the use of mobile applications such as the one developed for this study.

Finally, we cannot define the lower age limit at which SVV is normalised with respect to controls in preterm infants due to the sample size of the age ranges. A study with a larger sample size would be needed to define it.

## Conclusion

After the results obtained in this study, we conclude that in the age group of 7–10 years there is no further impairment in the perception of verticality attributable to prematurity in children with no neurodevelopmental disorders born very preterm or with very low weight at birth. Furthermore, the SVV values are indifferent to sex.

These results are very relevant when it comes to knowing the ethology of possible balance disorders in children with these characteristics; the fact of knowing the integrity of the vestibular system may have implications in the development of treatments that are more directed to treat motor and proprioceptive deficits.

## Data Availability

The data that support the findings of this study are available and can be provided by the corresponding author, based upon reasonable request.
